# Meta-Analysis of the Association between Mir-196a-2 Polymorphism and Cancer Susceptibility

**DOI:** 10.3969/j.issn.2095-3941.2012.01.012

**Published:** 2012-03

**Authors:** Huan Zhang, Yu-liang Su, Herbert Yu, Bi-yun Qian

**Affiliations:** 1 Department of Epidemiology and Biostatistics, Tianjin Medical University Cancer Institute and Hospital, Tianjin 300060, China; 2 Key Laboratory of Cancer Prevention and Therapy, Tianjin Medical University, Ministry of Education, Tianjin 300060, China; 3 Epidemiology Program, University of Hawaii Cancer Center, Honolulu 96813, HI, USA

**Keywords:** MIRN196 microRNA, human, polymorphism, neoplasms, meta-analysis

## Abstract

**Objective:**

MicroRNA plays a vital role in gene expression, and microRNA dysregulation is involved in carcinogenesis. The miR-196a-2 polymorphism rs11614913 is reportedly associated with cancer susceptibility. This meta-analysis was performed to assess the overall association of miR-196a-2 with cancer risk.

**Methods:**

A total of 27 independent case-control studies involving 10,435 cases and 12,075 controls were analyzed for the rs11614913 polymorphism.

**Results:**

A significant association was found between rs11614913 polymorphism and cancer risk in four genetic models (CT *vs.* TT, OR=1.15, 95%CI=1.05–1.27; CC *vs.* TT, OR=1.23, 95%CI=1.08–1.39; Dominant model, OR=1.17, 95%CI=1.06–1.30; Additive model, OR=1.08, 95%CI=1.01–1.14). In the subgroup analysis of different tumor types, the C allele was associated with increased risk of lung, breast, and colorectal cancer, but not with liver, gastric, or esophageal cancer. In the subgroup analysis by ethnicity, a significantly increased risk of cancer was found among Asians in all genetic models, but no associations were found in the Caucasian subgroup.

**Conclusions:**

The meta-analysis demonstrated that the miR-196a-2 polymorphism is associated with cancer susceptibility, especially lung cancer, colorectal cancer, and breast cancer among Asian populations.

## Introduction

Cancer, as a major public health problem, is reportedly one of the leading causes of death worldwide ^[^^1^^]^. Moreover, cancer is a very complex genetic disease, the mechanism of which has not been completely elucidated. Studies have suggested that cancer development results from gene–environment interactions ^[^^2^^]^. The presence of high-frequency low-penetrance susceptibility genes may be important in carcinogenesis.

MicroRNA (miRNA) are 21 to 24 nucleotide-long, single-stranded, non-coding RNA. These evolutionarily highly conserved miRNA play vital roles in the regulation of gene expression through mRNA cleavage or translational repression ^[^^3^^,^^4^^]^. To date, 678 human miRNA have been characterized; they regulate the expression of approximately one-third of human genes^[^^5^^,^^6^^]^. miRNA are possibly involved in many biological processes, including cell differentiation, proliferation, and apoptosis ^[^^7^^]^. Evidences also suggest that miRNA may play important roles in tumor development and prognosis ^[^^8^^,^^9^^]^.

Two types of single nucleotide polymorphisms (SNPs) were discovered in miRNA genes based on their location. One type is located in the mature regions of the miRNA and the other is located in the pre-miRNA regions. The former may directly regulate both the binding to target mRNA and pre-miRNA maturation, whereas the latter may only influence the maturation of pre-miRNA ^[^^10^^,^^11^^]^.

The SNPs in pre-miRNA have drawn increasing attention because they influence the maturation of miRNA, and they play potential roles in tumor development and progression^[^^12^^]^. In 2008, Hu et al. ^[^^13^^]^ identified a polymorphism in miR-196a-2 with a T to C change (rs11614913). Rs11614913 is located in the 3′ passenger (3p) strand mature sequence of miR-196a-2. This functional polymorphism is reportedly associated with the susceptibility of various tumors, including lung cancer ^[^^14^^,^^15^^]^ and breast cancer ^[^^16^^]^, and with lower survival rates of non-small cell lung cancer ^[^^13^^]^, gliomas ^[^^17^^]^, gastric cancer ^[^^18^^]^, gallbladder cancer ^[^^19^^]^, head and neck cancer ^[^^20^^]^, esophageal cancer ^[^^21^^]^, and hepatocellular carcinoma ^[^^22^^,^^23^^]^. Since then, many epidemiologic studies have examined the associations between SNPs and the risks of various cancers in diverse racial/ethnic populations. However, the results of these studies are inconclusive because of their small sample size or lack of replication. Therefore, this meta-analysis was employed to investigate the association of the miR-196a-2 rs11614913 polymorphism with cancer risk.

## Materials and Methods

### Literature search

A meta-analysis was conducted based on all case-control studies that examined the association between miR-196a2 rs11614913 polymorphism and cancer risk. The terms rs11614913, polymorphism, and cancer were used to search in the PubMed, EMBASE, Cochran library, and Web of Knowledge databases. The last search was completed on January 31, 2012. No language restrictions were imposed in the meta-analysis. Two independent investigators performed the search, Zhang H and Su YL. The studies that fulfilled the inclusion criteria were selected for evaluation. A manual search was also conducted through reviewing the references cited by the selected articles.

### Inclusion criteria

The inclusion criteria for our meta-analysis were as follows: (1) a case-control study design; (2) an association reported between miR-196a2 rs11614913 polymorphism and cancer risk; (3) number of subjects in each genotype available or sufficient allele frequencies for estimating odds ratio (OR), and 95% confidence interval (CI).

### Data extraction

Two independent investigators evaluated all the studies selected and extracted the data as shown in Table 1. When a discrepancy was found, a third investigator was invited for discussion until a consensus was reached. The extracted data included the name of the first author, year of publication, country origin, ethnicity, cancer type, source of controls (population- or hospital-based), total number of cases and controls, and the number of different genotypes in the respective cases and controls.

**Table 1:** Characteristics of studies included in the meta-analysis.

No.	Study	Year	Ethnicity	Cancer type	Source of controll†	Genotyping method	HWE control		Case				Control		
									TT	CT	CC		TT	CT	CC
1	Min KT ^[^^24^^]^	2011	Asian	colorectral	PB	PCR-RFLP	0.633		125	201	120		148	254	100
2	Zhang M ^[^25^]^	2011	Asian	breast	PB	PCR-RFLP	0.904		148	89	11		133	93	17
3	Zhan JF ^[^^26^^]^	2011	Asian	colorectral	HB	PCR-RFLP	0.849		56	128	68		163	267	113
4	Zhou B ^[^^27^^]^	2011	Asian	squamous cell cancer	HB	PCR-RFLP	0.077		57	123	46		82	169	58
5	Hong YS ^[^14^]^	2011	Asian	lung	HB	TaqMan	0.163		96	224	86		134	198	96
6	Zhang XW ^[^28^]^	2011	Asian	hepatocellular	PB	PIRA-PCR	0.52		277	449	208		477	817	328
7	Zhu LJ ^[^^29^^]^	2011	Asian	colorectral	NC	TaqMan	0.79		130	303	140		172	295	121
8	Chen H ^[^^30^^]^	2011	Asian	colorectral	HB	PCR-LDR	0.788		35	64	27		107	206	94
9	Okubo M ^[^31^]^	2010	Asian	gastric	HB	PCR-RFLP	0.51		166	281	105		223	350	124
10	Dou T ^[^^17^^]^	2010	Asian	glioma	HB	PCR-LDR	0.119		189	343	111		208	305	143
11	Wang K ^[^32^]^	2010	Asian	esophageal	NC	SNaPshot	0.6		48	262	148		111	250	128
12	Peng S ^[^^18^^]^	2010	Asian	gastric	HB	PCR-RFLP	0.936		43	94	76		50	107	56
13	Qi P ^[^^33^^]^	2010	Asian	hepatocellcular	NC	PCR-LDR	0.793		159	286	115		102	197	92
14	Tian T ^[^^15^^]^	2009	Asian	lung	PB	PCR-RFLP	0.7		293	512	253		307	519	209
15	Hu Z ^[^^34^^]^	2009	Asian	breast	PB	PCR-RFLP	0.207		287	483	239		358	517	218
16	Li XD ^[^^22^^]^	2010	Asian	hepatocellcular	HB	PCR-RFLP	0.402		82	150	78		78	102	42
17	Kim JM ^[^35^]^	2010	Asian	lung	PB	PCR-MCS	0.6		162	305	187		185	300	155
18	Jedlinski DJ ^[^36^]^	2011	Caucasion	breast	PB	PCR-RFLP	0.83		33	86	68		31	82	58
19	Akkiz H ^[^23^]^	2011	Caucasion	hepatocellular	HB	PCR-RFLP	0.492		22	86	77		40	87	58
20	Mittal RD ^[^37^]^	2011	Caucasion	bladder	HB	PCR-RFLP	0.003		5	131	76		14	127	109
21	George GP ^[^38^]^	2011	Caucasion	prostate	PB	PCR-RFLP	0.002		3	101	55		10	114	106
22	Liu Z ^[^^20^^]^	2010	Caucasion	head and neck	HB	PCR-RFLP	0.737		194	565	350		202	545	383
23	Srivastava K ^[^19^]^	2010	Caucasion	gallbladder	PB	PCR-RFLP	0.068		16	95	119		19	75	136
24	Catucci I ^[^39^]^	2010	Caucasion	breast	NC	TaqMan	0.708		244	842	766		377	1246	1116
25	Hoffman AE ^[^16^]^	2009	Caucasion	breast	NC	Sequenom MassARRAY	0.583		36	209	181		71	229	166
26	Ye Y ^[^^21^^]^	2008	Caucasion	esophageal	HB	SNPlex assay	0.477		83	141	83		59	172	107
27	Vinci S ^[^^40^^]^	2011	Caucasion	lung	NC	HRMA	0.267		12	54	35		10	61	58

†, HB:hospital-based; PB:population-based; NC:not clear.

### Statistical analysis

The consistency of the data with the Hardy–Weinberg equilibrium (HWE) was determined using a χ^2^ test after comparing the observed and expected genotypic frequencies in the control populations; a *P*-value <0.05 was considered statistically significant (Table 1).

The association between miR-196a-2 polymorphism and cancer risk was evaluated by calculating the pooled OR and 95% CI. A Z-test was used to determine the significance of the pooled OR, and *P*<0.05 was considered statistically significant. The meta-analysis examined the SNP associations in the following models: the co-dominant model (TT *vs.* CC and CT *vs.* CC); the dominant model (TT+CT *vs.* CC), and the recessive model (TT *vs.* CC+CT).

The χ^2^-based *Q* test was used to detect heterogeneity, and I^2^ index was adopted to measure the extent of heterogeneity ^[^^41^^,^^42^^]^. A *P*-value>0.10 ^[^^43^^]^ in the Q test indicates a lack of heterogeneity among the selected studies. If no heterogeneity was found, the Mantel–Haenszel method was used to estimate the pooled OR of all individual studies in a fixed-effect model ^[^^44^^]^. Otherwise the random-effect model (the DerSimonian and Laird method) ^[^^44^^]^ was used when heterogeneity was observed among the selected studies.

Publication bias was evaluated using the funnel plot wherein the standard error of log(OR) for each study was plotted against its log (OR). The asymmetry of a funnel plot was determined using the Begg’s rank correlation test ^[^^45^^]^. Furthermore, the Egger’s linear regression test was used ^[^^46^^]^ to measure the association between the mean effect estimate and its variance. Subgroup analyses were also performed according to the cancer types and ethnicity. If a cancer type was evaluated only by one study, this was grouped as “other cancer.” The ethnicity was categorized into Asian and Caucasian descent. Moreover, sensitivity analyses were conducted to examine the robustness of the results by excluding one study at a time and recalculating the combined OR and 95%CI of the remaining studies.

All of the analyses were conducted using the STATA software (version 11.0; StataCorp, College Station, TX) and Review Manager (version 5.0.0; The Cochrane Collaboration, Oxford, England), with two-sided *P*-values.

## Results

### Characteristics of the studies

**Figure 1:**
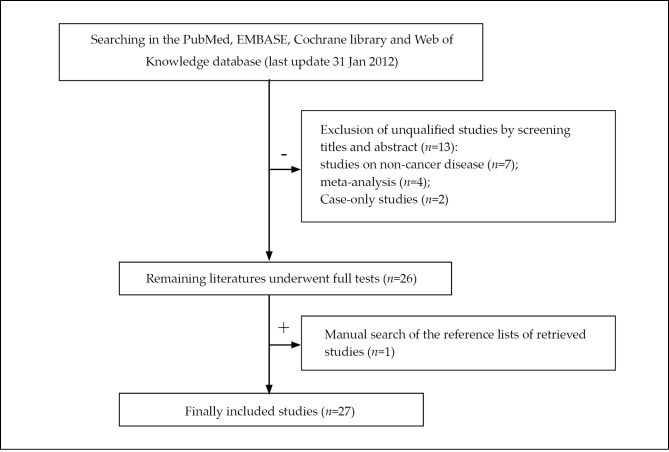
Flow diagram of the identification of studies.

A total of 39 articles were initially identified through database searches using different key words and their combinations. After reading the titles and abstracts of the identified articles, the studies that did not meet the inclusion criteria were excluded. During data extraction, additional articles that did not have allelic frequencies available for analysis were also disqualified. Eligible studies were retrieved for detailed full-text evaluation. Manual searches were also done and one relevant article from the references of selected studies was identified. Finally, 27 independent case-control studies ^[^^14^^-^^40^^]^, involving 10,435 cases and 12,075 controls, were included in the meta-analysis for rs11614913. Figure 1 shows the identification and selection of studies.

The characteristics of the eligible studies are summarized in Table 1. As shown in the table, 10 studies involved Caucasians and 17 involved Asians. Four studies focused on the association of SNPs with lung cancer, 4 with liver cancer, 5 with breast cancer, 4 with colorectal cancer, 2 with gastric cancer, 2 with esophageal cancer, and the remaining 6 studies were associated with other types of cancer. Up to 12 studies used hospital-based controls and 9 used population-based controls. The control sources for the other 6 studies were not identified clearly. The main genotyping method was polymerase chain reaction-restriction fragment length polymorphism (PCR-RFLP), which was used in 15 studies. The other genotyping methods employed included MassARRAY, Taqman, DNA sequencing, and high-resolution melting analysis. One study was published in 2008, 3 in 2009, 11 in 2010, and 13 in 2011.

### Quantitative synthesis

**Figure 2:**
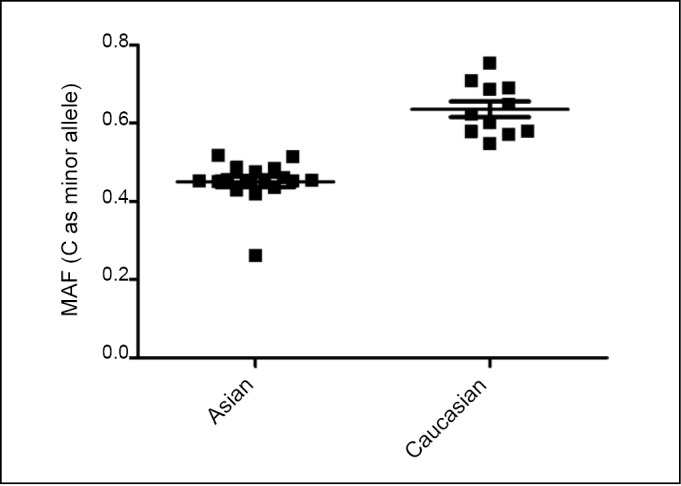
miR-196a-2 rs11614913 MAF distribution in different ethnicity.

Minor allele frequencies (MAF) were calculated in the control groups of each study to estimate the racial difference. Figure 2 shows the significant difference (C as the minor allele) in MAF distribution of miR-196a-2 rs11614913 T>C polymorphism among the controls of the different races: Asians and Caucasians. Specifically, the mean MAF in 17 Asian studies was 0.451±0.013. The mean MAF was 0.637+0.020 in the 10 Caucasian studies. The distribution of rs11614913 genotypes in the control subjects followed the HWE in all studies except 2, reported by Mittal et al. ^[^^37^^]^ and by George et al. ^[^^38^^]^ (Table 1).

### Meta-analysis results

The associations of the miR-196a-2 polymorphism with the risk of developing different cancer types among the different racial groups are shown in Table 2. In the meta-analysis of all studies, the rs11614913 polymorphism was significantly associated with the risk of cancer in 4 genetic models (CT *vs.* TT, OR=1.15, 95%CI=1.05–1.27; CC *vs.* TT, OR=1.23, 95%CI=1.08–1.39; Dominant model, OR=1.17, 95%CI=1.06–1.30; Additive model, OR=1.08, 95%CI=1.01–1.14).

**Table 2:** Combined ORs (95% CI) of subgroups for miR-196a-2 rs11614913.

Subgroup	No. of study	CC vs. TT		CT vs. TT		Dominant model^a^		Recessive model^b^		Additive model^c^	
		OR (95% CI)	Ph	OR (95% CI)	Ph	OR (95% CI)	Ph	OR (95% CI)	Ph	OR (95% CI)	Ph
Total	27	1.23(1.08-1.39)^r^	<0.00001	1.15(1.05-1.27)^r^	0.0002	1.17(1.06-1.30)^r^	<0.0001	1.06(0.97-1.16)^r^	<0.0001	1.08(1.01-1.14)^r^	<0.00001
Cancer Type											
Lung	4	1.26(1.07-1.49)^f^	0.26	1.15(1.00-1.33)^f^	0.13	1.19(1.04-1.36)^f^	0.21	1.07(0.85-1.35)^r^	0.07	1.12(1.03-1.22)^f^	0.15
Liver	4	1.31(0.87-1.95)^r^	0.006	1.11(0.87-1.43)^r^	0.08	1.19(0.88-1.61)^r^	0.01	1.17(0.91-1.49)^r^	0.07	1.14(0.93-1.40)^r^	0.006
Breast	5	1.23(0.92-1.65)^r^	0.01	1.11(0.98-1.25)^f^	0.13	1.15(0.93-1.42)^r^	0.03	1.10(1.00-1.21)^f^	0.15	1.09(0.96-1.24)^r^	0.02
Colorectrol	4	1.44(1.18-1.75)^f^	0.28	1.17(0.99-1.38)^f^	0.19	1.25(1.06-1.46)^f^	0.20	1.30(1.10-1.53)^f^	0.38	1.20(1.08-1.32)^f^	0.28
Gastric	2	1.24(0.94-1.65)^f^	0.31	1.07(0.85-1.34)^f^	0.85	1.12(0.90-1.39)^f^	0.70	1.22(0.96-1.55)^f^	0.16	1.12(0.98-1.28)^f^	0.23
Esophageal	2	1.22(0.26-5.72)^r^	<0.00001	1.19(0.29-4.81)^r^	<0.00001	1.20(0.28-5.11)^r^	<0.00001	1.05(0.63-1.74)^r^	0.02	1.05(0.55-2.00)^r^	<0.00001
Other	6	0.98(0.82-1.16)^f^	0.63	1.19(1.02-1.38)^f^	0.29	1.10(0.96-1.27)^f^	0.53	0.82(0.73-0.92)^f^	0.33	0.95(0.88-1.02)^f^	0.78
Ethnicity											
Asian	17	1.27(1.11-1.46)^r^	0.001	1.16(1.04-1.28)^r^	0.005	1.19(1.07-1.32)^r^	0.002	1.15(1.04-1.27)^r^	0.03	1.12(1.05-1.20)^r^	0.005
Caucasian	10	1.14(0.86-1.51)^r^	0.0005	1.18(0.92-1.51)^r^	<0.00001	1.16(0.90-1.50)^r^	0.0007	0.93(0.79-1.08)^r^	0.004	0.98(0.87-1.11)^r^	0.0004
Control Source											
PB	9	1.24(1.11-1.39)^f^	0.53	1.04(0.95-1.14)^f^	0.46	1.10(1.01-1.20)^f^	0.47	1.09(0.93-1.27)^r^	0.008	1.07(0.99-1.16)^r^	0.07
HB	12	1.18(0.96-1.47)^r^	0.0009	1.17(1.00-1.37)^r^	0.01	1.17(0.99-1.38)^r^	0.003	1.03(0.89-1.20)^r^	0.006	1.07(0.97-1.19)^r^	0.0008
NC	6	1.33(0.90-1.95)^r^	<0.00001	1.32(0.98-1.78)^r^	0.0003	1.32(0.96-1.83)^r^	<0.0001	1.09(0.92-1.29)^r^	0.03	1.11(0.95-1.31)^r^	<0.0001
Lab test method											
PCR-RFLP	15	1.27(1.15-1.41)^f^	0.12	1.11(1.02-1.21)^f^	0.35	1.16(1.07-1.25)^f^	0.24	1.10(0.95-1.26)^r^	0.0004	1.09(1.01-1.18)^r^	0.005
Others	12	1.14(0.92-1.41)^r^	<0.00001	1.17(0.98-1.39)^r^	<0.00001	1.16(0.97-1.39)^r^	<0.00001	1.03(0.92-1.16)^r^	0.01	1.06(0.96-1.16)^r^	<0.0001

Ph: *P*-value of Q-test for heterogeneity test; ^a^, Dominant model (CT+CC vs. TT); ^b^, Recessive model(CC vs. TT+CT); ^c^, Additive model(C vs. T); *f*, fixed model; *r*, random model.

The tumor type, race, control source, and lab test methods were adjusted as confounding factors in the overall analysis. Subgroup analysis was performed according to different tumor types, racial groups, control sources, and genotyping methods. In the subgroup analysis of tumor types, the C allele was associated with increased risks for lung cancer, breast cancer, and colorectal cancers, but was not associated with those for liver cancer, gastric cancer, and esophageal cancers. A significant association with lung cancer was observed in all genetic models (CC *vs.* TT, OR=1.26, 95%CI=1.07–1.49; CT *vs.* TT, OR=1.15, 95%CI=1.00–1.33; Dominant model, OR=1.19, 95%CI=1.04–1.36; Additive model, OR=1.12, 95%CI=1.03–1.22) except for the recessive model. Similarly, statistically significant associations were found for colorectal cancer in all models (CC *vs.* TT, OR=1.44, 95%CI=1.18–1.75; Dominant model, OR=1.25, 95%CI=1.06–1.46; Recessive model, OR=1.30, 95%CI=1.10–1.53; Additive model, OR=1.20, 95%CI=1.08–1.32). In breast cancer, the CC genotype was associated significantly with increased risk when compared with TT and CT genotype.

In the subgroup analysis of race, statistically significant associations with increased cancer risk were found among Asians in all genetic models. Specifically, the OR (95%CI) was 1.27 (1.11-1.46) for CC *vs.* TT, 1.16 (1.04-1.28) for CT *vs.* TT, 1.19 (1.07-1.32) for CT+CC *vs.* TT, 1.15 (1.04-1.27) for CC *vs.* CT+TT, and 1.12 (1.05-1.20) for C allele *vs.* T allele. However, no significant association was found for Caucasians in any of the genetic models.

In the subgroup analysis of the control source, the CC genotype was associated with cancer when the study used a population-based control (CC *vs.* TT, OR=1.24, 95%CI=1.11-1.39; Dominant model, OR=1.10, 95%CI=1.01-1.20). Nevertheless, the CT genotype was marginally associated with cancer risk in the hospital-based control subgroup (CT *vs.* TT, OR=1.17, 95%CI=1.00-1.37).

Subgroup analysis was also performed based on different genotyping methods. Statistically significant associations with increased cancer risk were found in the PCR-RFLP subgroup in all genetic models except for the recessive model. Specifically, OR (95%CI) was 1.27 (1.15-1.41) for CC *vs.* TT, 1.11 (1.02-1.21) for CT *vs.* TT, 1.16 (1.07-1.25) for CT+CC *vs.* TT, and 1.09 (1.01-1.18) for C allele *vs.*T allele. However, no significant association was found for genotyping methods other than PCR-RFLP in any of the genetic models.

### Test of heterogeneity

The heterogeneity of the studies was analyzed in the overall meta-analysis as well as subgroup analysis. The *P* values in the *Q* test were also shown in Table 2. The heterogeneity of the study decreased in the subgroup analysis of cancer type. If *P*>0.10, a fixed-effect model was chosen, otherwise a random-effect model was used.

### Sensitivity analysis

**Figure 3:**
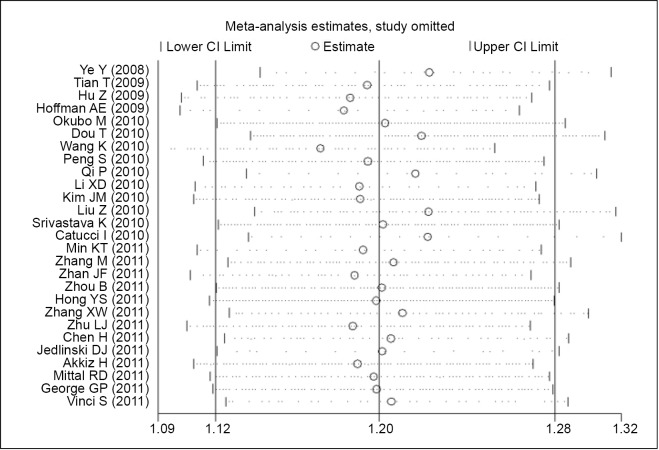
Pooled OR of sensitivity analysis.

In the sensitivity analysis, the meta-analysis was repeated by excluding 1 study each time to assess the influence of the removed study on the pooled ORs. The corresponding pooled ORs were not altered materially for rs11614913 (Figure 3 and Table 3).

**Table 3:** ORs (95% CI) of sensitivity analysis for rs11614913.

Omitting literature	CC vs. TT		CT vs. TT		Dominant model		Recessive model		Additive model	
	OR (95% CI)	Ph	OR (95% CI)	Ph	OR (95% CI)	Ph	OR (95% CI)	Ph	OR (95% CI)	Ph
All for rs11614913	1.23(1.08-1.39)	<0.00001	1.15(1.05-1.27)	0.0002	1.17(1.06-1.30)	<0.0001	1.06(0.97-1.16)	<0.0001	1.08(1.01-1.14)	<0.0001
Ye Y 2008 ^[^^21^^]^	1.26(1.12-1.42)	<0.0001	1.18(1.07-1.29)	0.003	1.20(1.09-1.32)	0.0005	1.07(0.98-1.18)	0.003	1.09(1.03-1.16)	<0.0001
Tian T 2009 ^[^^15^^]^	1.22(1.07-1.40)	<0.00001	1.16(1.05-1.29)	0.0002	1.18(1.06-1.31)	<0.00001	1.05(0.96-1.16)	<0.0001	1.07(1.01-1.14)	<0.00001
Hu Z 2009 ^[^^34^^]^	1.22(1.06-1.39)	<0.00001	1.16(1.04-1.28)	0.0002	1.17(1.05-1.31)	<0.0001	1.05(0.96-1.16)	<0.0001	1.07(1.01-1.14)	<0.00001
Hoffman AE 2009 ^[^^16^^]^	1.20(1.06-1.36)	<0.0001	1.14(1.03-1.25)	0.0006	1.15(1.05-1.27)	<0.0001	1.05(0.96-1.15)	<0.0001	1.07(1.00-1.13)	<0.00001
Okubo M 2010 ^[^^31^^]^	1.23(1.08-1.41)	<0.00001	1.16(1.05-1.29)	0.0001	1.18(1.06-1.31)	<0.00001	1.06(0.97-1.17)	<0.0001	1.08(1.01-1.15)	<0.00001
Dou T 2010 ^[^^17^^]^	1.25(1.10-1.42)	<0.00001	1.15(1.04-1.28)	0.0002	1.18(1.06-1.31)	<0.00001	1.08(0.99-1.18)	0.002	1.08(1.02-1.15)	<0.00001
Wang K 2010 ^[^^32^^]^	1.19(1.05-1.33)	0.0002	1.12(1.02-1.22)	0.01	1.14(1.04-1.24)	0.002	1.05(0.96-1.15)	<0.0001	1.06(1.00-1.13)	<0.0001
Peng S 2010 ^[^^18^^]^	1.22(1.07-1.39)	<0.00001	1.16(1.05-1.28)	0.0002	1.17(1.06-1.30)	<0.00001	1.05(0.96-1.15)	<0.0001	1.07(1.01-1.14)	<0.00001
Qi P 2010 ^[^^33^^]^	1.25(1.10-1.42)	<0.00001	1.17(1.05-1.29)	0.0002	1.19(1.07-1.32)	<0.0001	1.07(0.98-1.18)	<0.0001	1.08(1.02-1.15)	<0.00001
Li XD 2010 ^[^^22^^]^	1.21(1.06-1.38)	<0.00001	1.15(1.04-1.27)	0.0002	1.16(1.05-1.29)	<0.0001	1.05(0.96-1.15)	<0.0001	1.07(1.01-1.14)	<0.00001
Kim JM 2010 ^[^^35^^]^	1.22(1.07-1.39)	<0.00001	1.16(1.04-1.28)	0.0001	1.17(1.06-1.30)	<0.00001	1.05(0.96-1.16)	<0.0001	1.07(1.01-1.14)	<0.00001
Liu Z 2010 ^[^^20^^]^	1.24(1.09-1.42)	<0.00001	1.16(1.05-1.29)	0.0001	1.18(1.07-1.32)	<0.00001	1.07(0.98-1.18)	0.0001	1.08(1.02-1.15)	<0.00001
Srivastava K 2010 ^[^^19^^]^	1.23(1.08-1.40)	<0.00001	1.15(1.04-1.27)	0.0002	1.17(1.06-1.30)	<0.00001	1.08(0.98-1.18)	0.0001	1.08(1.02-1.15)	<0.00001
Catucci I 2010 ^[^^39^^]^	1.24(1.08-1.42)	<0.00001	1.16(1.05-1.29)	0.0002	1.18(1.06-1.32)	<0.00001	1.06(0.96-1.17)	<0.0001	1.08(1.01-1.15)	<0.00001
Min KT 2011 ^[^^24^^]^	1.22(1.07-1.39)	<0.00001	1.17(1.05-1.29)	0.0002	1.18(1.06-1.31)	<0.00001	1.05(0.96-1.15)	0.0001	1.07(1.01-1.14)	<0.00001
Zhang M 2011 ^[^^25^^]^	1.24(1.09-1.41)	<0.00001	1.17(1.06-1.29)	0.0003	1.19(1.08-1.32)	<0.0001	1.07(0.98-1.17)	<0.0001	1.08(1.02-1.15)	<0.00001
Zhan JF 2011 ^[^^26^^]^	1.21(1.06-1.38)	<0.00001	1.15(1.04-1.27)	0.0002	1.16(1.05-1.29)	<0.0001	1.05(0.96-1.15)	<0.0001	1.07(1.00-1.14)	<0.00001
Zhou B 2011 ^[^^27^^]^	1.23(1.08-1.40)	<0.00001	1.16(1.05-1.28)	0.0001	1.18(1.06-1.31)	<0.00001	1.06(0.97-1.16)	<0.0001	1.08(1.01-1.15)	<0.00001
Hong YS 2011 ^[^^14^^]^	1.22(1.07-1.40)	<0.00001	1.14(1.03-1.26)	0.0006	1.16(1.05-1.29)	<0.0001	1.07(0.97-1.17)	<0.0001	1.07(1.01-1.14)	<0.00001
Zhang XW 2011 ^[^^28^^]^	1.23(1.08-1.41)	<0.00001	1.17(1.06-1.30)	0.0001	1.19(1.07-1.32)	<0.0001	1.06(0.96-1.16)	<0.0001	1.08(1.01-1.15)	<0.00001
Zhu LJ 2011 ^[^^29^^]^	1.21(1.06-1.38)	<0.00001	1.14(1.03-1.27)	0.0003	1.16(1.05-1.29)	<0.0001	1.06(0.96-1.16)	<0.0001	1.07(1.01-1.14)	<0.00001
Chen H 2011 ^[^^30^^]^	1.24(1.09-1.41)	<0.00001	1.16(1.05-1.28)	0.0002	1.18(1.07-1.31)	<0.00001	1.07(0.97-1.17)	<0.0001	1.08(1.02-1.15)	<0.00001
Jedlinski DJ 2011 ^[^^36^^]^	1.23(1.08-1.40)	<0.00001	1.16(1.05-1.28)	0.0002	1.18(1.06-1.31)	<0.00001	1.06(0.97-1.16)	<0.0001	1.08(1.01-1.15)	<0.00001
Akkiz H 2011 ^[^^23^^]^	1.20(1.06-1.37)	<0.00001	1.14(1.04-1.26)	0.0003	1.16(1.05-1.28)	<0.0001	1.05(0.96-1.15)	<0.0001	1.07(1.01-1.13)	<0.00001
Mittal RD 2011 ^[^^37^^]^	1.22(1.07-1.39)	<0.00001	1.14(1.04-1.26)	0.0004	1.17(1.06-1.29)	<0.0001	1.08(0.98-1.18)	0.0001	1.08(1.02-1.15)	<0.00001
George GP 2011 ^[^^38^^]^	1.22(1.07-1.39)	<0.00001	1.15(1.04-1.27)	0.0003	1.17(1.06-1.29)	<0.00001	1.08(0.99-1.18)	0.0002	1.08(1.02-1.15)	<0.00001
Vinci S 2011 ^[^^40^^]^	1.24(1.09-1.41)	<0.00001	1.16(1.05-1.28)	0.0001	1.18(1.07-1.31)	<0.0001	1.07(0.98-1.17)	<0.0001	1.08(1.02-1.15)	<0.00001

Ph: *P* value of Q-test for heterogeneity test.

### Publication bias

**Figure 4:**
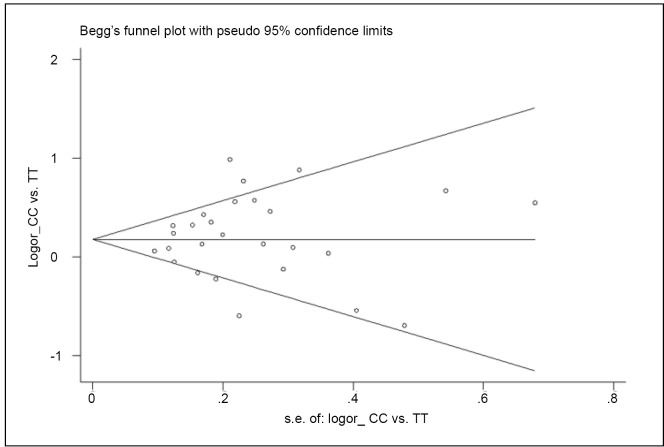
Begg’s funnel plot for rs11614913 (CC *vs.* TT).

Begg’s funnel plot and Egger’s test were performed to evaluate the publication bias of the study. The funnel plot for CC *vs.* TT of miR-196a-2 rs11614913 polymorphism seemed approximately symmetrical (Figure 4). The Egger’s test did not show any evidence of publication bias (*t*=0.60, *df*=26, *P*=0.553).

## Discussion

MiRNAs are important post-transcriptional regulators of gene expression and are reportedly involved in various diseases. Emerging miRNA profiling studies have investigated the differences in miRNA expression between cancer patients and healthy controls. Various miRNAs are upregulated or downregulated in different forms of tumors. However, the regulatory effects of miRNA in cancer occurrence and development are complicated. Although genetic and epigenetic regulation influence miRNA activity, the mechanism of miRNAs involvement in carcinogenesis remains unclear. Considering the extensive regulation of many target genes by miRNA, a single nucleotide polymorphism in the miRNAs gene may affect the expression and activity of various genes or proteins, which further influence the carcinogenic process and tumor progression.

A previous study found 323 SNPs located in 227 human pre-miRNA sequences ^[^^10^^]^. Another study integrated 474 human miRNA sequences, and found genetic variants located mainly in the precursor sequences (10%), whereas rare variants were located in the seed region (<1%) ^[^^35^^]^. Of these pre-miRNA SNPs, the miR-196a-2 rs11614913 T>C polymorphism is the most studied SNP. This functional polymorphism reportedly has an association with susceptibility to various forms of tumor including lung cancer, breast cancer, glioma, gastric cancer, gallbladder cancer, head and neck cancer, esophageal cancer, hepatocellular carcinoma, as well as shortened survival time in non-small cell lung cancer.

The results of the meta-analysis show that people carrying the variant genotype C have an increased risk of cancer compared to those carrying the wild genotype T. This finding coincides with a previous meta-analysis ^[^^47^^]^. The miR-196a-2 polymorphism may play a role in carcinogenesis.

Stratification of the analysis in terms of tumor type revealed that the miR-196a-2 polymorphism was related to lung cancer, colorectal cancer, and breast cancer, but no relationship was seen for liver cancer, gastric cancer, or esophageal cancer. The differences may be attributed to the following: miRNAs may have tissue-specific expression and the same miRNA SNP may play different roles in different tissues, which leads to different degrees of carcinogenesis; the sample size in each subgroup was not large enough to have sufficient study power to generate reliable results. However, our subgroup analysis showed substantial results in accordance with previous studies on various types of cancer. Previous meta-analyses has found that rs11614913 is associated with colorectal cancer risk ^[^^48^^]^, breast cancer risk^[^^49^^]^, and lung cancer risk ^[^^47^^]^, consistent with the results of our study.

In the subgroup analysis by race, an increased risk of cancer was found among Asians in all genetic models, but not among Caucasians. The distribution of the minor allele in the controls of these populations was also evaluated. Significant differences in the distribution of C allele were observed. Caucasians have a higher frequency of the C allele compared with Asians. This difference in allelic frequency may affect genetic susceptibility in different racial/ethnic groups.

In the subgroup analysis in terms of the control source, the C allele was associated with cancer susceptibility in studies with population-based controls. Moreover, the population-based subgroup was less heterogeneous than the hospital-based subgroup, which indicated that population-based controls are more helpful in reducing the heterogeneity of observational studies.

Finally, the C allele was associated with cancer risk among studies that used PCR-RFLP for genotyping. However, in subgroups using other genotyping methods, no significant association was found. Different genotyping methods may have different capacities for detecting gene polymorphism, which may influence the results. The laboratory test method may be one of the confounding factors in the overall analysis. After adjusting for genotyping method, the rs11614913 C genotype was associated with cancer risk.

Currently, two possibilities explain the association of the miR-196a-2 polymorphism with cancer risk. First, the rs11614913 variant may increase mature miR-196a expression and enhance target mRNA binding ^[^^13^^]^. Zhan et al. ^[^^26^^]^ reported that the miR-196a expression level among C allele carriers was higher than that in those carrying the TT genotype in colorectal cancer. Similarly, Li et al. found increased miR-196a expression among hepatocellular carcinoma patients with Hepatitis B virus infection who carried the C allele ^[^^22^^]^. This SNP is associated with G_2_ cell cycle delay, which plays a vital role in carcinogenesis^[^^17^^]^.

The current meta-analysis has several limitations. First, the cancer types included in the analysis were limited. Although the polymorphism and cancer risk in all reported studies were analysed, these studies mainly focused on several tumor types, which may affect the risk estimation. Second, some of the study controls were not from healthy populations. Selection bias could have occurred, which may have confounded the results. Third, the lack of individual information inhibited the calculation needed to adjust the ORs and analyze the gene-environment interaction.

In conclusion, miR-196a-2 polymorphism is associated with cancer risk, especially lung cancer, colorectal cancer, and breast cancer. Asians carrying the miR-196a-2 variant genotype are more susceptible to cancer compared with Caucasians. The SNP in miR-196a-2 may be a key factor in carcinogenesis. Furthermore, well-designed studies involving various ethnic populations and more cancer types are needed to confirm the results. Functional studies are needed to clarify the mechanisms that show the effect of miRNA SNPs on cancer development.
